# Measuring Thermal Diffusivity of Azoheteroarene Thin Layers by Photothermal Beam Deflection and Photothermal Lens Methods

**DOI:** 10.3390/ma16186312

**Published:** 2023-09-20

**Authors:** Ameneh Mikaeeli, Dorota Korte, Humberto Cabrera, Dariusz Chomicki, Dariusz Dziczek, Oksana Kharchenko, Peng Song, Junyan Liu, Andreas D. Wieck, Michal Pawlak

**Affiliations:** 1 Institute of Physics, Faculty of Physics, Astronomy and Informatics, Nicolaus Copernicus University in Torun, Grudziadzka 5, 87-100 Torun, Poland; chomicki@doktorant.umk.pl (D.C.); dziczek@umk.pl (D.D.); mpawlak@fizyka.umk.pl (M.P.); 2 Chair of Applied Solid-State Physics, Faculty of Physics and Astronomy, Ruhr-University Bochum, Universitaetsstrasse 150, D-44780 Bochum, Germany; andreas.wieck@ruhr-uni-bochum.de; 3 Laboratory of Enviromental Research, University of Nova Gorica, Vipavska 13, SI-5000 Nova Gorica, Slovenia; dorota.korte@ung.si; 4 MLab, STI Unit, The Abdus Salam International Centre for Theoretical Physics, Strada Costiera 11, 34151 Trieste, Italy; hcabrera@ictp.it; 5 Faculty of Chemistry, Taras Shevchenko National University of Kyiv, 64/13 Volodymyrska St., 01601 Kyiv, Ukraine; oksana_kharchenko@ukr.net; 6 MiNt Laboratory, University of Angers, 4 Rue Larrey, 49100 Angers, France; 7 School of Instrumentation Science and Engineering, Harbin Institute of Technology, Harbin 150001, China; pengsong@hit.edu.cn (P.S.); ljywlj@hit.edu.cn (J.L.); 8 State Key Laboratory of Robotics and System, Harbin Institute of Technology, Harbin 150001, China

**Keywords:** thin films, photothermal spectroscopy, thermal transport, thermal wave, thermal conductivity, thermal diffusivity

## Abstract

Measurement of thermal properties of thin films is challenging. In particular, thermal characterization is very difficult in semi-transparent samples. Here, we use two photothermal methods to obtain information about the thermal diffusivity as well as thermal conductivity of azoheteroarene functionalized polymer thin layers. The photothermal beam deflection (PBD) method is employed to gather data directly on thermal conductivity and thermal diffusivity, while the thermal lens (TL) method is employed to measure the effective thermal diffusivity. Consequently, the thermal diffusivity of the layers is indirectly estimated from the effective thermal diffusivity using a well-established theoretical relationship. Despite the utilization of distinct methods, our study reveals a remarkable consistency in the highly accurate results obtained from both approaches. This remarkable agreement reaffirms the reliability and mutual compatibility of the employed methods, highlighting their shared ability to provide accurate and congruent outcomes.

## 1. Introduction

Photothermal methods are a class of high-sensitivity characterization methods that have been applied to determine the photothermal parameters of liquid and solid samples in particular thin films [1]. The underlying principle of the method revolves around the generation of thermal waves by selectively heating the surface of the sample and/or its surrounding region using a modulated laser beam. This controlled heating induces changes in the physical characteristics of the sample. Photothermal techniques, in essence, rely on the creation of temperature gradients within the material and its immediate environment, which are closely related to the fundamental thermal properties of the samples, encompassing thermal conductivity, thermal diffusivity, and thermal boundary resistance. The main advantages of photothermal techniques are their contactless nature, localized heating, as well as high sensitivity and accuracy due to the direct measurement of the deposited heat that the optical absorption causes on the sample [1,2,3,4,5]. The photothermal effect depends on the opto-thermal properties of the sample under examination. Numerous methods have been employed to ascertain and analyze the thermal behavior of multilayer structures. For instance, photothermal infrared radiometry and thermoreflectance [6,7] have been widely used by researchers. However, when dealing with thin films, the low absorbance of these materials often requires more sensitive methods to achieve accurate results. Moreover, the propagation of thermal waves in thin films is influenced by both the thermal diffusivity and the excitation frequency (thermal penetration depth). Therefore, frequency-domain methods can be employed to selectively confine the generated heat within the specific region of interest for measurement. In this context, thermal lens spectroscopy (TLS), which was first introduced by Gordon et al. in 1965 [8], has been utilized for semi-transparent material measurements. TLS offers significant advantages for assessing semi-transparent thin films, including its high sensitivity due to direct measurement of nonradiative energy transfer. Additionally, TLS enables the measurement of photothermal properties, including absorption, thermal conductivity, and thermal diffusivity [9,10]. On the other hand, photothermal beam deflection spectroscopy (PBDS) has proven its efficacy in characterizing opaque and weakly absorbing samples [11,12,13,14,15,16]. Among the available non-contact experimental techniques, frequency-domain methods are particularly preferred for several reasons. Firstly, they enable depth profilometry. Secondly, they provide two channels of information (amplitude and phase). Finally, these methods leverage lock-in detection for enhanced sensitivity and accuracy.

The transfer of heat in insulating polymers such as azoheteroarene functionalized poly (methyl methacrylate) occurs through the diffusion of vibrational modes, a process that strongly relies on the arrangement of macromolecules within the polymer structure. Consequently, any modification to the macromolecular ordering (adding electron-donating or electron-withdrawing substituents to the side chain) possesses the potential to profoundly alter the thermal transport properties of the polymer. Azobenzene is a class of π-conjugated compounds that features two or more phenyl rings linked by an azo (–N=N–) bridge [17]. It was first studied in 1834 for its ability to change its conformation when exposed to light. A distinctive characteristic of azobenzene lies in its capacity to assume two isomeric forms, *trans* and *cis*, and the invertible photoisomerization reaction between them, allowing interconversion between these two isomers. The *trans*→*cis* isomerization occurs under UV-visible light with a wavelength of 320 nm–350 nm (π→π* transition), while the *cis*→*trans* isomerization happens spontaneously in the dark or when exposed to light with a wavelength of 400 nm–450 nm (n→π* transition). Azoheteroarenes are azobenzene derivatives in which one or two phenyl rings were substituted with other aromatic moieties. They are appealing due to their simple structure, low-cost production technology, and fascinating physicochemical properties as compared to traditional materials. This has led to their use in next-generation optical limiters, molecular memory storage, organic photoconductors, and nonlinear optical elements [18].

In this paper, we propose TLS and PBDS as two complementary methods for measuring the thermal diffusivity of three different azo-dye functionalized polymer thin films [19,20] deposited on glass substrate using spin coating method. Hence, the first method enables the determination of the effective thermal diffusivity of the bulk sample (azo + glass), subsequently facilitating the estimation of the thermal diffusivity of the individual layers. Effective thermal diffusivity considers the thermal properties and geometry of composite materials. It considers factors such as the individual thermal diffusivities and conductivities of the constituent materials as well as their volume fractions, in order to extract value of thermal diffusivity of the layers. The second method makes it possible to obtain information from different areas of the sample using the modulation frequency, which allows the investigation of thin films. Both methods offer an enhanced signal-to-noise ratio in comparison to time-domain and steady-state techniques.

## 2. Materials and Methods

### 2.1. Thermal Lens

**Figure 1:**
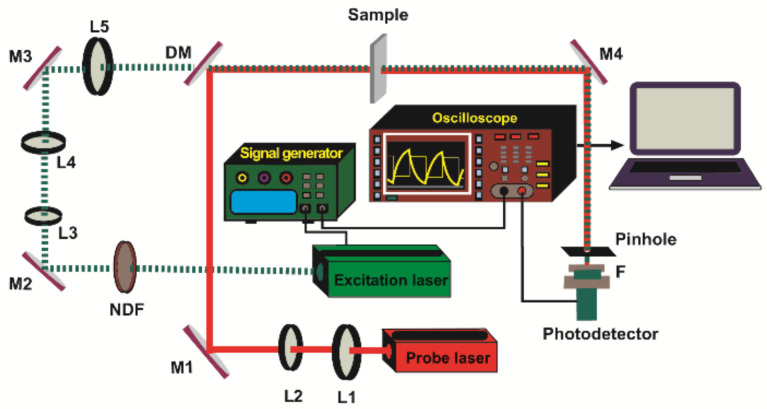
Configuration of the experimental setup utilized to measure the thermal effective diffusivity in polymer samples. The optical components include lenses (L1–L5), turning mirrors (M1–M4), a dichroic mirror (DM), a neutral density filter (NDF), and a filter (F).

The scheme of the thermal lens setup for measuring the thermal diffusivity of polymer samples is shown in Figure 1. The magnitude of the heat produced and distributed within the sample stems from the outcomes of non-radiative de-excitation processes of the absorbed radiation from the excitation beam (EB) taking place in the examined sample. As a source of EB, a modulated laser beam with Gaussian intensity distribution is usually used. The produced heat introduces the temperature gradients in the sample, known as TL. As the refractive index is influenced by temperature variations, there occurs a spatial distribution of the index to an extent similar to the generated heat in the absorbing sample. 

The detection of the generated TL involves the utilization of a probe beam (PB), which is another laser beam in the setup. This TL carries essential information regarding the characteristics of the sample under examination [10]. The excitation process employs a 405 nm diode-pumped solid-state laser, which is modulated at 2 Hz through a signal generator. To ensure optimal conditions, the excitation beam undergoes collimation using lenses L3 and L4 before being precisely focused onto the specimen using lens L5. The PB, sourced from a He-Ne laser (632.8 nm, 3 mW), is carefully collimated by means of lenses L1 and L2. Both the EB and the PB beams are collinearly directed through the sample using mirrors M1, M2, and M3, with a dichroic mirror (DM) facilitating this process. The changes in the PB intensity are quantified using a Si detector, equipped with a pinhole of 0.7 mm diameter. Direct acquisition of the TL signal is performed using a digital oscilloscope. Further insights into the signal processing and fitting procedure can be accessed from a separate reference [9].

### 2.2. Photothermal Beam-Deflection Spectroscopy

**Figure 2:**
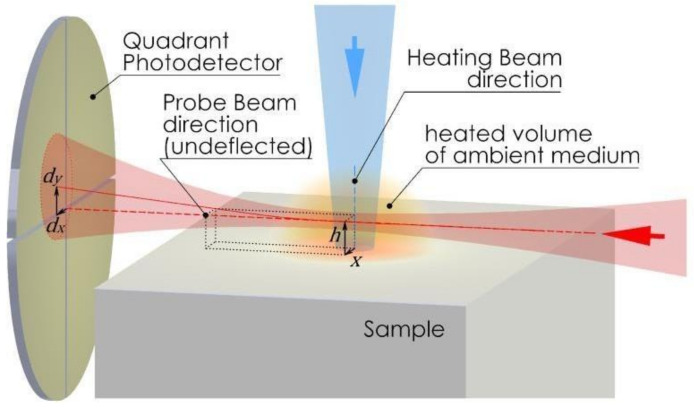
PBDS experimental setup for detecting RICs and RIGs. The probe laser beam (PB) grazes the sample via a transparent medium, influenced by RIGs and RICs. Detection is achieved using a quadrant photodetector (QPD).

The fundamental premise governing photothermal beam-deflection spectrometry (PBDS) involves illuminating the sample’s surface with modulated excitation radiation (ER) in a perpendicular orientation. The sample absorbs the incident light energy, which is further converted into heat due to non-radiative relaxation processes. As a result of heat transfer, periodical temperature oscillations (TOs) are induced in both the sample and the adjustment medium close to its surface, creating changes in the fluid refraction index (RICs) and its gradients (RIGs). There exist three types of PBDS experiments: transverse, collinear, and mixed. Transverse measurements (TPBDS) are performed on opaque samples, whereas collinear (CPBDS) and mixed (MPBDS) ones are used for transparent samples. In the context of the experimental arrangement in a TPBDS configuration, the detection of RICs and RIGs involves a probe laser beam (PB) that grazes the sample surface via a transparent medium (Figure 2), which is positioned in direct contact with the solid surface subjected to irradiation by the pump beam at normal incidence. In terms of CPBDS, the PB propagates through the sample parallel to ER, whereas in case of MPBDS, it hits the sample at a certain angle, crossing ER within the examined sample. The PB is then deflected by RIGs, and its phase is changed as a consequence of RICs, which leads to changes in the PB’s intensity, which are detected by a quadrant photodetector (QPD). The collected signal is dependent on the thermo-optical properties of the sample (thermal diffusivity, conductivity, and absorption coefficient). Thermal property assessment in PBDS experiments is achieved through a comparative analysis. This involves contrasting the measured phase delay curve based on the relative positions of the PB and the ER with the calculated phase delay curve derived from a range of theoretical analyses encompassing various thermal property values [11,12,13,14,15,16].

All these methods suffer from some limitations of their applicability. These are related to the finite size of the ER as well as the non-zero value of the height of the PB over the sample surface, which introduces a shift in thermal parameter measurement, specially observed when the thermal properties of the sample are much lower than those of the fluid in which the PB propagates. Furthermore, the phase slope technique holds validity when considering substantial PB-ER offsets (Figure 2) in relation to the size of the PB spot and low modulation frequencies of ER, ensuring the characteristic length of the heterogeneous nature of the material LCH (related to the size of its grains) is smaller than the spatial range of the thermal disturbance μ_th_ ≈ 2πμ, where µ is the thermal diffusion length.

**Figure 3:**
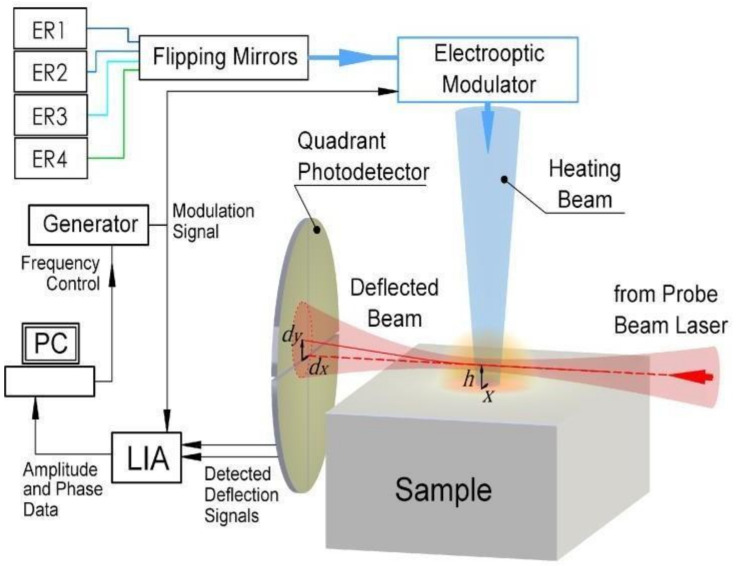
Experimental setup for PBDS method: an ER source guided by dielectric mirrors. The focused ER interacts with samples, generating TOs detected by a He-Ne laser (632.8 nm), captured via a PB system, and measured using a QPD. Data processing involves a lock-in amplifier (LiA) and PC.

The choice for the ER source was deliberated from the category of diode-pumped solid-state lasers emitting 375 nm, 445 nm, 532 nm, and 785 nm, depending on the absorbance of the examined sample and optimized experimental conditions. The tunability regarding ER was achieved by changing the position of flipping mirrors (Figure 3). The ER was modulated using an electro-optic modulator connected to a signal generator that provided modulation frequencies ranging from DC to GHz, which ensured high through-plane resolution and data collection from thin sample layers (10 s nm). To avoid interaction with the support material, the modulation frequency range was selected in a way that the thermal diffusion length was comparable or less than the depth where the information was required to be collected. A set of mirrors (UV dielectric mirror, 1.0 in diameter, 300–550 nm; VIS dielectric mirror, 1.0 in diameter, 450–700 nm; NIR dielectric mirror, 1.0 in diameter, 700 nm–900 nm, Newport) directed the ER perpendicularly to the sample surface and focused it to a spot radius of about 50 µm using a lens with a focal length of 100 mm. TOs induced in the fluid above the sample were detected by the PB from a He-Ne laser with a 632.8 nm output wavelength and 2 mW output power. The PB was collimated and focused onto the sample’s surface into a spot with a 40 µm diameter using a series of lenses with focal lengths of 40 mm and 100 mm. The intensity variation of the PB was measured using QPD, which was equipped with an interference filter (632.8 nm CWL). This setup was connected to a lock-in amplifier (LiA) and a PC for data acquisition and processing. The sample was placed on a 3D translation stage, which allowed the experimental arrangement to be optimized. The sample was also moved with ER in relation to the PB frequency, allowing the sample through-plane thermal properties to be determined. All measurements were performed in air at room temperature.

### 2.3. Azoheteroarene Functionalized Polymer Thin Films

**Figure 4:**
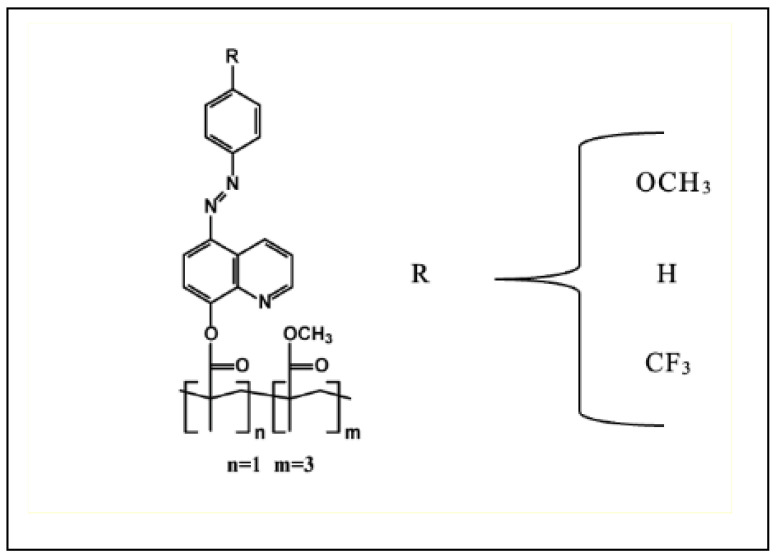
The chemical structure of the studied copolymers with various electron-donating and electron-withdrawing substituents.

The chemical structure of the studied copolymers is shown in Figure 4. Synthesis of the studied copolymers and their molecular weights were reported by Chomicki et al. [21]. The azo-dyes used to functionalize the polymers are based on 8-hydroxy quinoline, which is an electron-deficient moiety with a basic nitrogen [22]. They are attached to the side-chain of the copolymer, resulting in an atactic statistical copolymer with a functionalized:non-functionalized methyl methacrylare monomer ratio (n:m) equal to 1:3. Various electron-donating and electron-withdrawing substituents are attached to the para position of the phenyl ring of the chromophore. This modification results in changes in the thermal and optical properties of the prepared samples. The list of substituents attached to the phenyl ring in various polymers is given in Table 1.

The deposition of copolymers onto glass substrates (2.5 cm × 2.00 cm) was carried out using the spin-coating technique (Spin Coater SCC-200, Novocontrol Technologies). Prior to the deposition process, the glass substrates underwent a thorough cleaning procedure involving ultrasonic treatment with detergent, followed by sequential rinsing with DMF and acetone. Between each cleaning step, the substrates were meticulously rinsed with deionized water. Subsequently, droplets of 50 g/L polymer solutions were cast onto immobile substrates and then spun (2040 rpm, 60 s).

## 3. Signal Modeling

### 3.1. Thermal Lens Theory

According to the theory developed in [9,10], the TL signal S(*z*,*t*) is defined as follows:

(1) $${S(z,t)=\Theta \tan}^{-1}\left\{\frac{4m(z)\nu (z)t/t_{c}\left(z\right)}{\left[1+2m\left(z\right)+\nu {\left(z\right)}^{2}\right]2t/t_{c}\left(z\right)+{\left[1+2m\left(z\right)\right]}^{2}{+\nu (z)}^{2}}\right\},$$

where *t_c_* is TL characteristic time, $t_{c}=\omega_{0e}^{2}/4D$, and Θ is given by

(2) $$\Theta =\frac{P_{e}\alpha l}{\lambda_{p}\kappa }\frac{\mathrm{dn}}{\mathrm{dT}}$$

where P_e_ is the sample’s excitation power, and α, $\frac{\mathrm{dn}}{\mathrm{dT}}$, and l are the sample’s absorption coefficient, the temperature coefficient of the optical path, and the sample thickness. The thermal conductivity κ=ρc.D is related with the thermal diffusivity through the density and heat capacity.

The parameter $m={\left(\omega_{p}/\omega_{e}\right)}^{2}$ embodies a factor of mode-mismatching between the radii of the excitation and probe beams, while ν(*z*) constitutes the geometrical factor as follows:

(3) $$\nu (z)=\frac{{z-a}_{p}}{z_{p}}+\frac{z_{p}}{L-z}\left[1+\frac{{\left({z-a}_{p}\right)}^{2}}{z_{p}^{2}}\right]$$

where *z* is the sample position; *L* denotes the distance spanning from the sample to the detector; $a_{p}$, $z_{p}$, and $\lambda_{p}$ are the waist positions, Rayleigh parameter, and wavelength of the probe beam, respectively.

### 3.2. Photodeflection Signal

One of the signals is perpendicular to the sample surface (*S_PDn_*) and is called the normal component of PDS, whereas the other is parallel to the sample surface (*S_PDt_*) and is called the tangential component of PDS. The *S_PDn_* is derived from the difference in illumination between the upper and lower QP halves [23]:

(4) $$S_{PDn}=K_{d}\left(\int_{0}^{+\infty }-\int_{-\infty }^{0}dz_{D}\;\right)\int_{-h}^{+\infty }dy_{D}I\left(x_{D},y_{D},z_{D}\right),$$

whereas *S_PDt_* is the difference between the left and right QP halves:

(5) $$S_{PDt}=K_{d}\int_{-h}^{+\infty }dx_{D}\left(\int_{0}^{+\infty }-\int_{-\infty }^{0}dy_{D}\;\right)I\left(x_{D},y_{D},z_{D}\right),$$

where *K_d_* symbolizes the detector’s constant, and *h* denotes the height of the PB over the sample. Equations (4) and (5) incorporate the consideration that QPD is partially covered by the sample. In our case, the ER (of around 3 mm in diameter on the sample surface) is much wider than the PB (of around 50 μm in diameter in the area of TOs). In such a case, the tangential component of PDS can be neglected, since the TOs can be assumed to be 1D.

## 4. Experimental Results and Discussion

**Figure 5:**
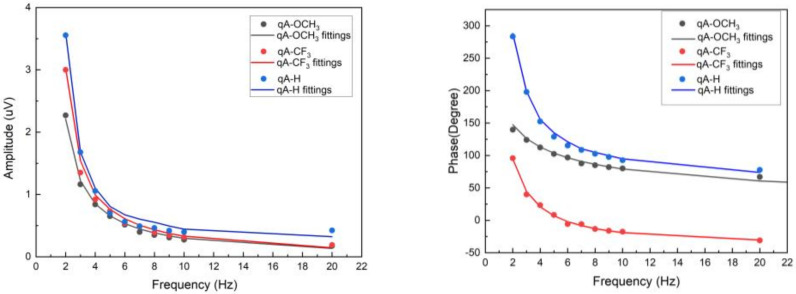
Best fits of the PBDS model to the experimental data of the studied samples qA-CF_3_, qA-H, and qA-OCH_3_.

Figure 5 shows the experimental data and the best fit using the theoretical models (Equation (5)) of PBDS for the amplitude and phase of the signal.

The experimental results obtained by PBDS for the study samples (qA-H, qA-CF_3_, and qA-OCH_3_) are in good agreement with the theory described with the theoretical model. The difference between experimental and theoretical data may also be the result of the assumed thickness value of 500 nm for all layers. Knowing the exact thickness of the sample could result in better fitting and lower errors.

In comparison with the PBDS data obtained for $t_{l}$, it appears that the thickness of the layer is not needed for analysis. It follows that TLS can be a good solution for determining layers of unknown thickness. Then, the determined thermal diffusivity can be used as a known parameter, and the thermal conductivity of a thin layer can be determined knowing the thermal diffusivity, with the unknown parameter as the thickness.

**Figure 6:**
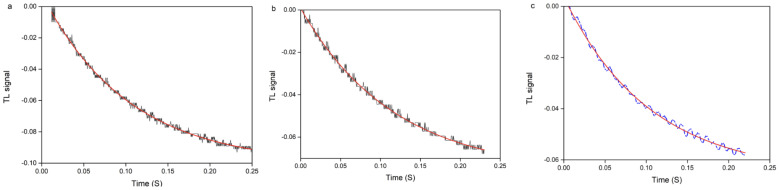
The experimental TL time-dependent signal of samples (**a**) qA-CF_3_, (**b**) qA-H, and (**c**) qA-OCH_3_, fitted with the theoretical TLS model (red line) using Equation (1).

In the case of thin layers, the TL method measures the effective thermal diffusivity parameters. From this value, one can calculate the thermal diffusivity of the thin layer. Figure 6a (qA-CF_3_), Figure 6b (qA-H) and Figure 6c (qA-OCH_3_) represent the experimental TL time-dependent signal for the studied samples, fitted with Equation (3) and using, as fitting parameters, Θ and D within the time interval ranging from T0 = 0 to Tf = 0.25 s. By considering the influence of various substituents on the thermal properties of polymers by taking qA-H as a reference because of its neutral characterization, we can see that the electron-withdrawing CF_3_ enhances the TL signal. The influence of various substituents on the thermal properties of the copolymers is considered in relation to the qA-H sample bearing the neutral hydrogen atom. In this regard, we can see that the electron-withdrawing CF_3_ substituent enhances the TL signal. Conversely, the electron-donating OCH_3_ group decreases the TL signal compared to H. These results provide insight into the role of substituents in modulating the thermal behavior of the material and emphasize the electron-withdrawing group’s prominent influence.

**Figure 7:**
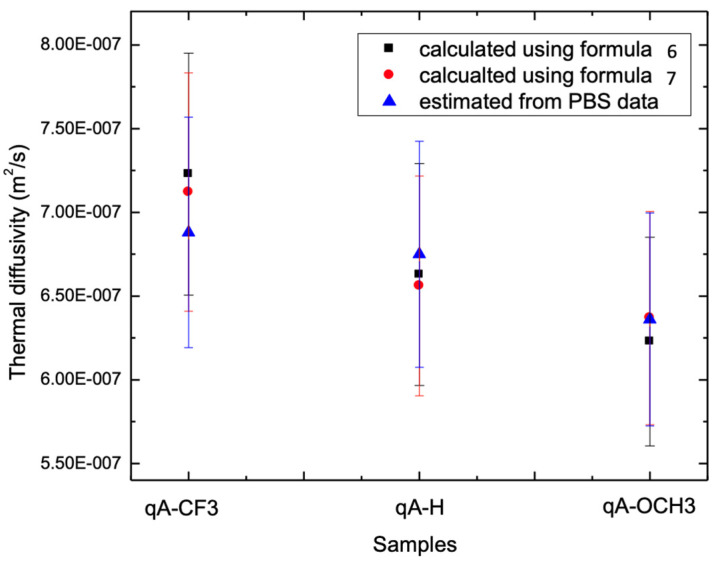
Comparison of calculated thermal diffusivity of azobenzene thin films (qA-OCH_3,_ qA-H, qA-CF_3_) using the TL method (Equations (6) and (7)) and measured using the PBDS method.

Figure 7 shows the comparison of the calculated thermal diffusivity of a thin layer from the effective thermal diffusivity using the following two equations and the PBDS method.

(6) $$\alpha_{1}=\left(\frac{1}{L_{1}}\right)\left(\alpha_{eff}\left(L_{1}+L_{2}\right)-L_{2}\alpha_{2}\right)$$

(7) $$\alpha_{1}=\frac{\alpha_{eff}\left(x^{2}\right)+\left(1-x\right)\lambda }{1-\frac{\alpha_{eff}{\left(1-x\right)}^{2}}{\alpha_{2}}+\alpha_{eff}\left(1-x\right)\frac{1}{\lambda }\sigma_{2}}$$

According to Equations (6) and (7), $L_{1}+L_{2}$, $x=\frac{L_{1}}{L}$, and $\lambda =\frac{k_{1}}{k_{2}}$, where $k_{1}$ and $k_{2}$ are the thin film and glass substrate thermal diffusivity, $\alpha_{2}$ is the glass substrate thermal diffusivity, and *α_eff_* is the measured effective thermal diffusivity. For calculation, $k_{2}\;$= 1.011 W/mK, $\alpha_{2}\;$= 0.44 × 10^−6^ (m^2^/s), and *L*_1_ = 500 nm. The calculated values are shown in Table 2.

Table 2 also presents the error of estimation parameters. For PBDS, the errors associated with thermal conductivity, thermal diffusivity, and the optical absorption coefficient stem from the fitting procedure. Conversely, for the thermal lens method, a distinct error calculation approach is employed. Notably, the measured error of effective thermal diffusivity, as depicted in Figure 6, is considerably minimal. However, the reported errors for thin films in Table 2 exhibit larger magnitudes due to indeterminate thickness (as indicated in Formulas (6) and (7)).

As one can see, the values of thermal diffusivity calculated from the effective thermal diffusivity of azoheteroarene functionalized polymer samples measured using TLS agree well, within the measurement error (~10%), with the values obtained from PBDS.

While Figure 7 displays a discernible trend of diminishing thermal diffusivity, these variations remain confined within the margins of the imprecision of the determination of thermal diffusivity. A more precise understanding of this phenomenon necessitates more accurate measurements. Consequently, we have plans to undertake these comprehensive investigations in forthcoming studies.

## 5. Conclusions

We measured the thermal diffusivity and the effective thermal diffusivity in composite materials composed of azo-dye functionalized polymer and glass. The effective thermal diffusivity takes into account both the thermal properties and geometry of the composite materials. It considers factors such as the individual thermal diffusivities and conductivities of the constituent materials, as well as their volume fractions, in order to extract the thermal diffusivity value of the composite layers. In our study, we demonstrated that while the TLS method offers an effective means to measure the effective thermal diffusivity, it is a highly sensitive technique, particularly when applied in transparent samples. Nevertheless, the results obtained using the TLS method exhibited excellent agreement with those obtained from PBDS, further validating the reliability and accuracy of both methods in determining the thermal diffusivity. This suggests that experimental techniques like TLS and PBDS can be used as preliminary and complementary tools for thin film characterization.

## Figures and Tables

**Table 1:** Electron-donating and electron-withdrawing substituents attached to the side chain of polymers.

Type of Substituent	Electron-Donating	Neutral	Electron-Withdrawing
Substituent	qA-OCH_3_	qA-H	qA-CF_3_

**Table 2:** Measured and calculated thermal diffusivity of the investigated samples.

Sample	Determined Using PBDS			Determined Using TLS		
	$k_{1}$ (W/mK)	$\alpha_{1}$ (m^2^/s) × 10^−6^	Optical Absorption Coefficient (1/cm) × 10^3^	$\alpha_{eff}$ (m^2^/s) × 10^−6^	$\alpha_{1}$ (m^2^/s) × 10^−6^ Using Formula (6)	$\alpha_{1}\;(m^{2}/s)\;\times \;10^{-6}$ Using Formula (7)
qA-CF_3_	1.06 ± 0.09	0.69 ± 0.07	40 ± 10	0.86 ± 0.08	0.72 ± 0.07	0.71 ± 0.07
qA-H	1.04 ± 0.09	0.68 ± 0.07	58 ± 20	0.83 ± 0.08	0.66 ± 0.07	0.67 ± 0.07
qA-OCH_3_	0.97 ± 0.09	0.64 ± 0.06	56 ± 20	0.81 ± 0.08	0.62 ± 0.06	0.64 ± 0.06

## Data Availability

Data is available upon request to the corresponding author.
